# Everybody's Page

**Published:** 1889-06-01

**Authors:** 


### Everybody's Page.

The directors of an American railway of the "slow and sure" order, boasted that there had never been an accident on it. "No"; remarked a critic—a shareholder in an opposition line—"but once a middle-aged gentleman left one station on it, and died of old age before he reached the next."

Chaulmugra oil is coming largely into use in veterinary surgery. It is beneficial in open sores, wounds, sprains, and rheumatic complaints, and is especially useful in sores caused by the friction of harness, to which horses that have to journey long distances are almost inevitably liable. The Government has adopted it, and it is now used in the cavalry and artillery regiments.

It seems rather strange to hear of an English missionary being anything else than a Christian one. But Colonel Olcott, who has just gone to Japan, and seems to be welcomed there, is an apostle of Buddhism, and is exhorting the natives to cling to the Buddhist creed, and not to yield it up for the teachings of either Christianity or modern science. But do Colonel Olcott and his listeners mean the same thing? We doubt if the popular creed of Japan is identical with the esoteric Buddhism, brought up to date, of imaginative Europeans.

Whoever has visited Northwich or any other centre of the salt industry would be inclined to say at a first glance "this town is intoxicated." For the houses lurch over to one side or another as if in their humble way they were all trying to be leaning towers of Pisa. The roads are uneven, and the whole district is full of holes and depressions of more or less magnitude. This is due to the subsidence of the land as the salt is mined away from beneath it; and the inhabitants are now trying to get a Bill brought into Parliament to settle the difficult question of the compensation due to proprietors for the injury brought by this land subsidence.

This is an age of advertisement; let us pay honour to the advertiser. But is that an altogether wise aspirant to such glory who offers the Government £20,000 for the privilege of advertising on the back of postage-stamps. Nobody really likes licking a stamp, and the literature that underlay the gum would be regarded with either indifference or abhorrence. Then there is the flavour of printer's ink to be added to the not particularly appetising flavour of the mucilage. Disguised with rose or vanilla we might endure it; but printer's ink plus gum, *au naturel*, would be too much, and the result would be a run on those ingenious rollers for moistening the back of stamps. In which case we should affix them without seeing the advertisement at all.

In the State of New Jersey there is a spicemill in which a great proportion of the work done is in the way of adulteration. When mustard has been crushed out of the seeds, the shells of these are ground and used to adulterate pepper. Meanwhile the mustard is not sent into the market in its natural state, but is mixed with cornmeal, turmeric, and red pepper. It is possible, indeed, to turn out a "mustard" with no mustard in it, but only a miscellaneous collection of adulterants made pungent with cayenne pepper. Ginger is adulterated with cornmeal, cabin biscuit, and cayenne. Cloves are ground up with 50 per cent. of charred walnut-shells, and the bulk of cinnamon is increased by admixture with cocoanut-shells. In each case a small flavour of cayenne is added to make up for any lack of pungency in the nutshells. These mixtures are not sold directly to the public by the manufacturing firm; they only supply the trade.

Some, even of the most simple foods, are injurious to certain people. Prof. Annandale, of Edinburgh, declares that in exceptional cases mutton has been known to act as poison.

Some time ago an American, who was staying in Egypt, found a few grains of wheat in the wrapp ngs of a mummy 5,000 years old. He planted them and managed to raise thirteen good ears. These he sent to a relative in Oregon, who is now trying to raise grain from them. It would be strange if "mummy wheat" were ever to come into the market.

Women are pushing their way into every department of work. The lady journalist, though a creature of recent birth, is beginning to make her presence felt on this side of the water; but she is much more *en evidence* on the other side of the Atlantic, where two graduates of Vassar, the American Girton, bought the property of a dying newspaper, revived its drooping circulation, and not only do all the editorial work connected with it, but manage a large printing establishment.

The sanitary state of Melbourne is causing great anxiety. During the last eighteen years there have been 3,816 deaths from typhoid, and typhoid is distinctly a preventible disease. The desirability of the compulsory notification of disease is shown by the fact that since an Act to that effect came into use the number of cases reported to the authorities has been nearly three times as many as those admitted before—1,725 as against 652—but the death-rate from typhoid has decreased. Secresy is one of the opportunities of disease. When a case is reported to the authorities there is a much better chance of precautionary and remedial measures being taken.

A new vegetarian journal has appeared in Berlin, and in it Dr. Eduard Reich traces all the ills, moral and physical, that flesh is heir to, to ailments of the stomach, which, of course, are due to improper food. But some of us have tried a vegetarian regimen—with results distinctly detrimental to comfort of body and peace of mind. Why is this? Either because we have not persevered with the treatment, or because our ancestors have so pampered their digestive organs that ours have inherited a weakness that unfits them for the severe simplicity of vegetarianism. The fathers have eaten sour grapes—or, in this case, beefsteak—and the children's teeth are set on edge. We submit to fate, and only ask that the steak shall be juicy and tender.

We have not many medical poets—Dr. Darwin, of "Botanic Garden" fame, and D. M. Moir, the biographer of Mansie Waugh, are perhaps the chief—but to the brief list should now be added the name of Dr. Kirkitar, a native practitioner of Bombay, who presented to the Australian Medical Congress the following sonnet—well intended, if not exactly of the highest merit:

"Ministers of health, who ceaseless, nobly fight
'Tween life and death, 'tween health disease and pain,
Shedding perennial, glorious, radiant light
Upon Hygeia's world-wide blest domain!
Free from this flow of reason—feast replete—
Go back to sacred duty, oh, but meet again,
And let me share your wisdom, mine's the gain—
When in this solemn conclave ye may meet.
Live long, Australian *confrères*, live and work,
Not with the lurid eye that loves the gold;
Live as ye have, the glory of your race,
To cheer the hearts where pain and anguish lurk,
Your names will e'er these passing joys enfold,
And find within my heart a lasting place."

